# Temperature Effects on the Efficacy of *Phytoseiulus persimilis* and *Amblyseius swirskii* (Acari: Phytoseiidae) Against *Tetranychus urticae* (Acari: Tetranychidae) in Strawberry Crops

**DOI:** 10.3390/insects17040366

**Published:** 2026-03-29

**Authors:** Lassaad Mahmoud Mdallel, Abderrahman Mquiteb, Abdallah Guerban, Bader Sulaiman Sudayri, Selman Al-Oudah, Soltan Mohamed Al-Eid

**Affiliations:** 1 National Organic Agriculture Center, Department of Protection and Biological Control, Ministry of Environment, Waiter and Agriculture, Unaiza 51911, Saudi Arabia; 2 Saudi Organic Farming Association, Riyadh 11321, Saudi Arabia

**Keywords:** two-spotted spider mite, phytoseiid predators, biological control, greenhouse temperature, predator release strategy

## Abstract

The two-spotted spider mite, *Tetranychus urticae* Koch, is a major arthropod pest that causes substantial economic losses in strawberry and other crops worldwide. Its management often relies on intensive acaricide applications, which may negatively affect human health, the environment, and beneficial organisms. Biological control using predatory mites has been widely adopted under greenhouse conditions, and its success is influenced by several factors, particularly temperature, *T. urticae* population density, and predator release methods. During the 2023–2024 season, this study evaluated the effects of ambient greenhouse temperature on the population dynamics of *T. urticae* and on the efficacy of two phytoseiid predators, *Phytoseiulus persimilis* (Athias-Henriot) and *Amblyseius swirskii* (Athias-Henriot). The predators were released individually and in combination on strawberry plants. The efficacy of single and combined releases was assessed under three temperature ranges: 25–27 °C, 28–30 °C, and 30–32 °C. The results showed that the mean number of motile *T. urticae* per plant increased from 21.7 to 95.66, while the mean number per leaf increased from 2.33 to 6.05 during the experimental period. The mean greenhouse temperature ranged from 23.83 °C to 31.88 °C. Temperature was strongly and positively correlated with *T. urticae* population density, with higher temperatures generally accelerating development and population growth. Both predator species, whether released individually or in combination, significantly reduced *T. urticae* populations compared with untreated control plants. *Amblyseius swirskii* demonstrated greater efficacy than *P. persimilis* at temperatures above 28 °C. However, the combined release consistently achieved the highest level of mite suppression across all temperature ranges. Significantly greater population reduction was observed at approximately 25–26 °C compared with single-species releases, while maximum effectiveness occurred at temperatures above 28 °C. These findings indicate that the combined use of *P. persimilis* and *A. swirskii* represents an effective biological control strategy for managing *T. urticae* in greenhouse-grown strawberries, particularly under elevated and fluctuating temperature conditions.

## Simple Summary

*Tetranychus urticae* Koch (Acari: Tetranychidae) is a globally important mite pest that causes substantial economic losses in strawberry production. *Phytoseiulus persimilis* (Athias-Henriot) and *Amblyseius swirskii* (Athias-Henriot) are among the most effective predatory mites used in biological control programs. Temperature is a key abiotic factor influencing the population dynamics of both pest and predatory mites. This study demonstrates that elevated temperatures accelerate the development of *T. urticae*, increase its population density, and affect the performance of predatory mites. Moreover, the combined release of *P. persimilis* and *A. swirskii* under ambient greenhouse temperatures was more effective than single-species releases. Therefore, the combined release of *P. persimilis* and *A. swirskii* may represent an effective strategy for controlling *T. urticae* on greenhouse strawberries and mitigating the effects of temperature variability.

## 1. Introduction

The two-spotted spider mite, *Tetranychus urticae* Koch (Acari: Tetranychidae), is a pernicious pest affecting numerous greenhouse crops, particularly strawberries [1,2]. The larvae, nymphs, and adults of *T. urticae* damage plants by using their piercing–sucking mouthparts to extract chlorophyll from leaf tissues. This feeding activity reduces the photosynthetic capacity of the plant, leading to significant yield losses and increased susceptibility to pathogens and viruses [3,4]. In addition, *T. urticae* infestation can reduce the nutritional quality of strawberries and decrease their market value [2,5].

Several factors influence the population growth parameters of *T. urticae*, including developmental rate, survival, reproduction, and longevity. These parameters are affected by host plant species, nutritional quality, cultivar, phenological stage, pesticide exposure, relative humidity, and temperature [6,7,8,9]. Elevated temperatures and dry conditions shorten the development time from egg to adult in *T. urticae* and increase female fecundity [10]. Consequently, such conditions may intensify damage to strawberry crops [11].

The conventional management of *T. urticae* in strawberries primarily relies on the application of acaricides [12,13]. However, excessive use of these chemicals has led to the development of resistance in *T. urticae*, environmental contamination, and adverse effects on human health and beneficial organisms [14,15,16]. Therefore, the development of environmentally friendly pest control strategies is essential.

Biological control methods including organic acaricides, plant extracts, natural oils, entomopathogens, and predatory insects and mites represent eco-friendly alternatives [3,17,18]. Among predatory mites, *Phytoseiulus persimilis* Athias-Henriot, *Stethorus punctillum* Weise, *Neoseiulus californicus* (McGregor), and *Amblyseius swirskii* Athias-Henriot (Acari: Phytoseiidae) are highly voracious and effective predators of *T. urticae* [2,11,19,20,21,22]. *Phytoseiulus persimilis* has been successfully used to control *T. urticae* populations in various greenhouse crops, including cucumbers, tomatoes, and strawberries, in regions such as the Mediterranean basin, Japan, Egypt, Turkey, and New Zealand [2,19,23,24,25]. *Stethorus punctillum* is also a major predator of *T. urticae* and other spider mites [26,27]. It is widely distributed across temperate regions of North America, Europe, and Asia [26]. Both its larval and adult stages consume eggs, larvae, and adults of *T. urticae*, making it an effective biological control agent [26,27]. *Amblyseius swirskii* is a generalist predator widely employed in biological control programs. It is commonly used to manage thrips and whitefly populations in greenhouse systems and can also contribute to the suppression of *T. urticae* and eriophyid mites [28,29].

The dispersal and efficacy of predatory mites such as *P. persimilis, S. punctillum*, and *A. swirskii* are influenced by several factors, including temperature, plant species and architecture, release timing, and application methods. Temperature plays a critical role, as increasing temperatures generally enhance developmental rates, searching efficiency, and predation rates up to an optimal threshold; however, beyond this point, their survival and efficacy may decline [27,28,29,30]. Plant species and architecture also influence predator performance. Due to their small size, predatory mites must traverse plant surfaces to locate prey, and the allocation of time among moving, resting, and feeding activities significantly influences their effectiveness [28].

Predatory mite efficacy is also affected by release strategies. In Integrated Pest Management (IPM) programs targeting *T. urticae*, *P. persimilis* may be released alone [2,30] or in combination with other predators such as *S. punctillum*, *N. californicus*, and *A. swirskii* [31,32,33,34,35]. The combination of two or more natural enemies in IPM programs can enhance pest suppression, particularly when predators target different pest life stages or employ complementary feeding strategies. This approach may lead to stronger and more reliable pest control and contribute to a more stable and balanced agroecosystem [36,37,38]. However, successful implementation of multiple predators in IPM programs requires several conditions, including the availability of preferred prey, suitable environmental conditions, limited interspecific competition, appropriate light intensity, supplementary food sources when necessary, and optimal temperature ranges for each predator species [39,40,41,42].

Previous studies have shown that the optimal temperature varies among predatory mite species [43,44,45,46,47,48,49,50]. For example, *P. persimilis*, considered one of the most specialized biological control agents against *T. urticae* on greenhouse strawberries, shows reduced efficacy at temperatures above 30 °C and when relative humidity falls below 60% [47,48].

In countries with arid and semi-arid climates, temperature fluctuations during winter and spring pose significant challenges for greenhouse strawberry production. Periods of unusually high or low temperatures may reduce the efficacy of *P. persimilis* when temperatures exceed 30 °C and diminish the performance of *A. swirskii* when temperature falls below 25 °C. Therefore, this study aims to determine whether the combined release of *P. persimilis* and *A. swirskii* can enhance the biological control of the two-spotted spider mite on strawberry plants under greenhouse conditions. Specifically, the study investigates the effect of temperature on *T. urticae* population dynamics and evaluates the efficacy of *P. persimilis* and *A. swirskii* when released individually and in combination.

## 2. Materials and Methods

### 2.1. Greenhouse Experiment

An experiment was conducted from November 2023 to May 2024 in a greenhouse at the National Organic Agriculture Center in Unaiza, Al-Qassim, Saudi Arabia. The greenhouse, measuring 40 m in length and 9 m in width, was divided into 15 plots prior to planting. Each plot measured 12 m^2^ and was completely covered with an ultrafine insect-proof net to prevent mite movement between treatments. Strawberry seedlings (cultivar ‘Festival’) were obtained from a commercial nursery in Unaiza, Al-Qassim, and transplanted on 24 December 2023 into 30 cm diameter pots filled with a soil mixture consisting of two-thirds peat moss and one-third sand. For each plot, nine plants were used, with one plant per pot. The pots were spaced 1 m apart within rows and 1 m between rows. Plants were manually irrigated four times per week.

### 2.2. Temperature and Humidity Data

At the beginning of the experiment, HOBO MX1101 temperature (Onset Computer Corporation, Bourne, MA, USA) and relative humidity data loggers (USA) were installed to record the daily temperature and relative humidity inside the greenhouse. In addition, strawberry plants were examined using a DM Wi-Fi digital microscope (TOMLOV, Shenzhen, China) (1000×; Ver. 1.9, Build 21) to confirm the absence of mites.

### 2.3. Experimental Design

The experiment was arranged in a randomized complete block design (RCBD) with three replications and four treatments. The treatments were: (1) a single release of *P. persimilis*, (2) a single release of *A. swirskii*, (3) a combined release of *P. persimilis* and *A. swirskii*, and (4) an untreated control. A total of 108 strawberry plants were used in the experiment. Each treatment consisted of 27 plants, with nine plants per replicate across the three replications.

### 2.4. Development of T. urticae Population on Strawberry Plants

On 22 January 2024, all strawberry plants, each bearing an average of eight to ten leaves, were artificially infested with *T. urticae*. The mites originated from infested strawberry leaves collected from a separate greenhouse. For each pot, a single leaf harboring at least seven motile stages (deutonymphs and adults) was placed onto a healthy, uninfested plant. The mites subsequently dispersed from the introduced leaf to the new host plant. From 29 January to 15 May 2024, the *T. urticae* population was monitored in an untreated plot comprising 27 strawberry plants. The total number of motile stages per plant and per leaf was recorded. Counts were conducted directly on each leaf using a 1000× DM Wi-Fi digital microscope (Ver. 1.9, Build 21) without detaching the leaves. A correlation analysis was performed to examine the relationship between *T. urticae* population density and the prevailing temperature [51].

### 2.5. Experiment Execution and Recorded Data

In early February 2024, predatory mites (*P. persimilis* and *A. swirskii*) were obtained from the Biocontrol and Bumblebee Production Center (Unaizah, Al-Qassim, Saudi Arabia). Upon arrival, the mites were examined to ensure their survival and activity, and were released into the greenhouse within 12 h. Prior to the application of treatments, twenty leaves were randomly selected from nine plants within each plot. The number of motile stages of *T. urticae* was counted using a Digital Microscope 1000× DM Wi-Fi. Predatory mites were released on two dates (5 and 25 February 2024) as follows: (1) *P. persimilis* at an approximate ratio of 1:10 (one predator per ten *T. urticae* individuals); (2) *A. swirskii* at an approximate ratio of 1:10; and (3) a combination of *P. persimilis* and *A. swirskii* at a (1 + 1):10 ratios (one individual of each predator species per ten *T. urticae* individuals). Strawberry plants in the untreated control group were left without predator release.

Data collection began seven days after the initial release and continued weekly until 15 May 2024. Each week, fifteen plants per treatment were randomly selected, and the total number of motile stages of *T. urticae* per plant was counted using the Digital Microscope 1000× DM Wi-Fi. The effectiveness of *P. persimilis*, *A. swirskii*, and their combination was evaluated under prevailing temperature conditions by calculating the percentage decrease (PD) in the *T. urticae* population density using the following formula:

PD (%) = [(NCdn1 − NTdn1)/(NCdn1)] × 100

where

NCdn1 = Number of spider mites per plant on untreated control plants.
NTdn1 = Number of spider mites per plant after application of a given treatment.
d = Date of collection.

### 2.6. Data Analysis

The effectiveness data were analyzed using a one-way analysis of variance (ANOVA). When significant differences were detected, treatment means were separated using Tukey’s post hoc test. All statistical analyses were performed using SPSS software (version 22). Differences were considered statistically significant at *p* ≤ 0.05. During the study period, Pearson correlation coefficients were calculated to evaluate the relationship between the number of motile *T. urticae* individuals and temperature, as well as between the effectiveness of predatory mites and temperature.

## 3. Results

### 3.1. Development of T. urticae Population on Strawberry Plants

**Figure 1:**
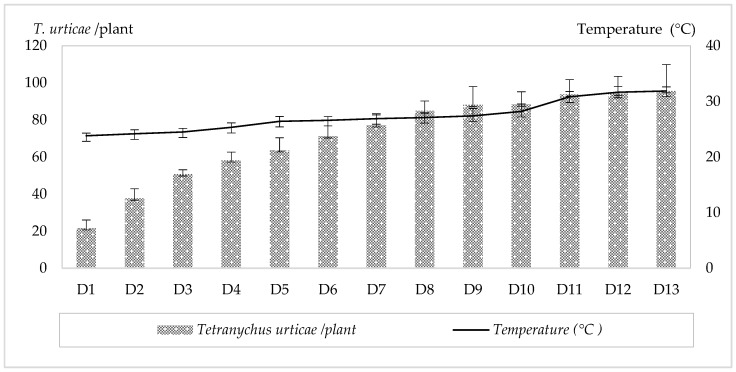
Weekly counts of *T. urticae* individuals per strawberry plant and the corresponding temperature during the experiment. (D1–D13: Weekly counts of motile *T. urticae* from the first observation date (D1) to the end of the experiment (D13)).

Populations of *T. urticae* on strawberry plants were monitored from 29 January to 15 May 2024. Following infestation, mite densities increased progressively over the 13-week observation period. The mean number of motile *T. urticae* per plant increased from 21.7 ± 4.33 (D1) to 95.66 ± 14.25 (D13), while the mean number per leaf increased from 2.33 ± 1.33 (D1) to 6.05 ± 1.33 (D13). During the same period, the average weekly temperature increased from 23.83 ± 0.48 °C to 31.88 °C. The weekly number of motile *T. urticae* per plant and the corresponding temperatures are shown in Figure 1. A highly significant positive correlation was observed between temperature and the *T. urticae* population on strawberry plants (r = 0.921, *p* = 0.0074). The linear regression equation describing this relationship was Y = 5.77X + 30.78 (R^2^ = 0.912), indicating that temperature explained 91.2% of the variation in mite population density.

### 3.2. Dynamic of T. urticae Population Under Different Treatments

**Figure 2:**
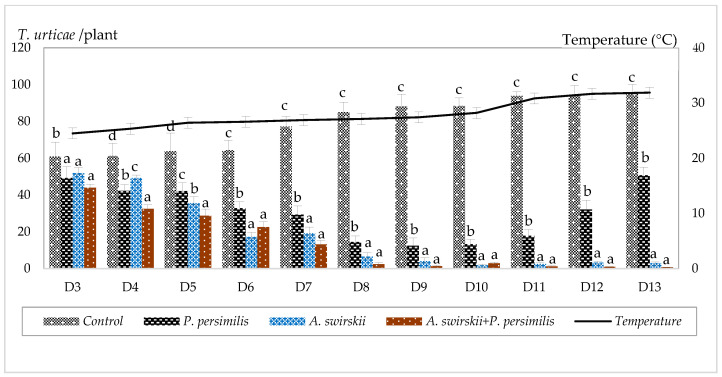
Dynamic of *T. urticae* populations on strawberry plants under different treatments at different dates. (D3–D13: sampling date after application of the treatments; different lowercase letters indicate significant differences among the treatments and control according to Tukey’s test at *p* < 0.05).

Figure 2 illustrates the population dynamics of motile *T. urticae* on strawberry plants under different treatments. In the control treatment, the number of motile mites per plant increased from 60.8 ± 7.95 (D3) to 95.66 ± 4.33 (D13). Following the release of *P. persimilis*, the population decreased from 49.24 ± 6.24 (D3) to 12.34 ± 4.26 (D9), before increasing again to 50.69 ± 5.38 by D13. In the treatment with *A. swirskii*, the population declined steadily from 51.94 ± 2.88 (D3) to 2.91 ± 0.63 (D13). A pronounced reduction in *T. urticae* density was observed following the combined release of *P. persimilis* and *A. swirskii*, where the population decreased from 43.49 ± 3.86 (D3) to 0.63 ± 0.24 (D13).

Weekly monitoring revealed significant differences among treatments. At D3, when the temperature was 24.5 ± 0.66 °C, no significant differences were detected among treatments 1, 2, and 3 (*p* = 0.27). At D4 (25.33 ± 0.82 °C), the lowest *T. urticae* population was observed in the combined-release treatment, which differed significantly from the single-release treatments of *P. persimilis* and *A. swirskii* (*p* = 0.001). At D5 (26.42 ± 0.88 °C), the highest population (63.8 ± 9.86) was recorded in the control treatment, whereas the lowest population (28.71 ± 3.66) occurred in the combined-release treatment. Intermediate population levels were recorded in the treatments with *A. swirskii* alone (35.61 ± 3.48) and *P. persimilis* alone (42.1 ± 4.46). These differences were statistically significant (*p* = 0.02). From D6 to D13, when temperatures exceeded 26.6 ± 0.96 °C, the highest *T. urticae* populations were consistently recorded in the control treatment, while the lowest populations were observed in the combined-release treatment. Populations in the *A. swirskii* single-release treatment were slightly higher than those in the combined-release treatment, although the difference was not significant. However, both treatments (*A. swirskii* alone and the combined release) resulted in lower *T. urticae* populations than the *P. persimilis* single-release and control treatments. Highly significant differences (*p* = 0.000) were recorded between the combined-release and *A. swirskii* single-release treatments compared with the *P. persimilis* single-release and control treatments.

### 3.3. Effect of Temperature on the Efficacy of P. persimilis to Control T. urticae on Strawberry

**Figure 3:**
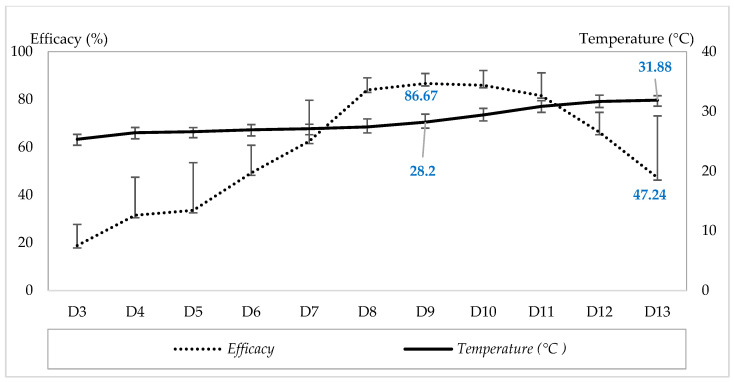
Efficacy of *P. persimilis* in controlling *T. urticae* on strawberry plants at different ambient temperatures. (D3–D13: Sampling dates after application of the treatments).

Figure 3 illustrates the efficacy of *P. persimilis* in controlling *T. urticae* on strawberry plants under a range of greenhouse ambient temperatures. During the experimental period (D3–D13), control efficacy ranged from 18.34 ± 2.38% to 86.67 ± 4.19%, while temperatures fluctuated between 25.33 ± 0.82 °C (D3) and 31.88 ± 0.74 °C. The highest efficacy (86.67 ± 4.19%) was recorded at 28.2 ± 1.34 °C. Within this temperature range, control efficacy increased with increasing temperature, showing a highly significant positive correlation (r = 0.925, *p* = 0.0081), as described by the regression equation Y = 12.05X + 4.12 (R^2^ = 0.90). However, at temperatures above 28.2 °C, the efficacy of *P. persimilis* declined, reaching 47.24% on D13 at 31.88 °C. In this higher temperature range, efficacy showed a significant negative correlation with temperature (r = −0.81, *p* = 0.046), as described by the regression equation Y = −9.85X + 103.1 (R^2^ = 0.85).

### 3.4. Effect on Temperature on Efficacy of A. swirskii to Control T. urticae on Strawberry in Greenhouse

**Figure 4:**
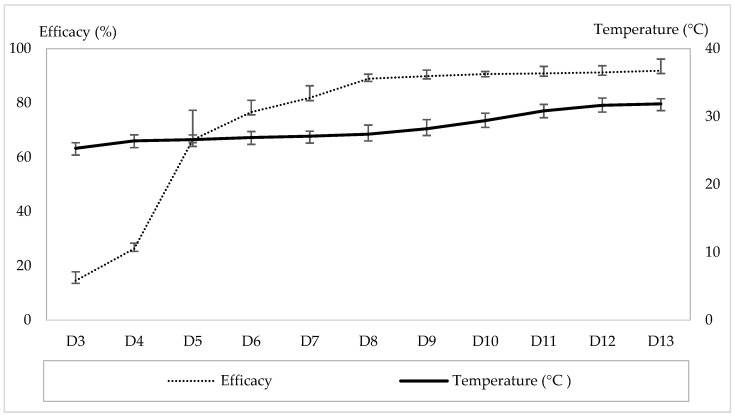
Efficacy of *A. swirskii* in controlling *T. urticae* on strawberry plants at different ambient temperatures. (D3–D13: Sampling dates after application of the treatments).

Figure 4 illustrates the efficacy of *A. swirskii* in controlling *T. urticae* on strawberry plants across a range of greenhouse ambient temperatures. Over the 10-week survey period, efficacy ranged from 14.56 ± 3.21% to 91.88 ± 4.32%. Efficacy increased with temperature, with the lowest value recorded at 25.33 ± 0.82 °C and the highest at 31.88 °C. This positive relationship was supported by a highly significant correlation (r = 0.925, *p* = 0.008). The relationship was described by the regression equation Y = 6.86X + 32.35 (R^2^ = 0.68).

### 3.5. Efficacy of Combining the Predators P. persimilis and A. swirskii to Control T. urticae on Strawberry in Greenhouse

**Figure 5:**
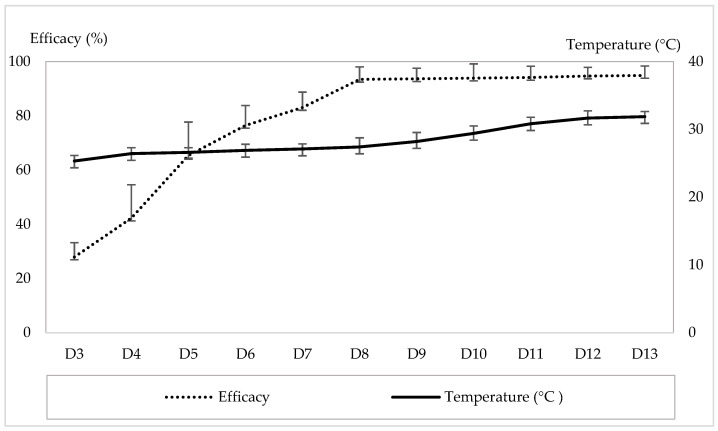
Efficacy of the combined release of *P. persimilis* and *A. swirskii* in controlling *T. urticae* on strawberry plants across a range of ambient temperatures. (D3–D13: Sampling dates after application of the treatments).

Figure 5 illustrates the efficacy of the combined release of *P. persimilis* and *A. swirskii* in controlling *T. urticae* on strawberry plants under a range of greenhouse ambient temperatures. Over the ten weekly post-treatment assessments, efficacy ranged from 27.92 ± 5.95% to 94.88 ± 4.32%. Efficacy increased with increasing temperature, with the lowest value recorded at 25.33 ± 0.82 °C and the highest at 31.88 °C. This positive relationship was further supported by a highly significant correlation (r = 0.69, *p* = 0.0093), as described by the regression equation Y = 6.14X + 41.26 (R^2^ = 0.75).

### 3.6. Comparative Efficacy of P. persimilis, A. swirskii, and Their Combined Use for Controlling T. urticae Across a Range of Temperatures

**Figure 6:**
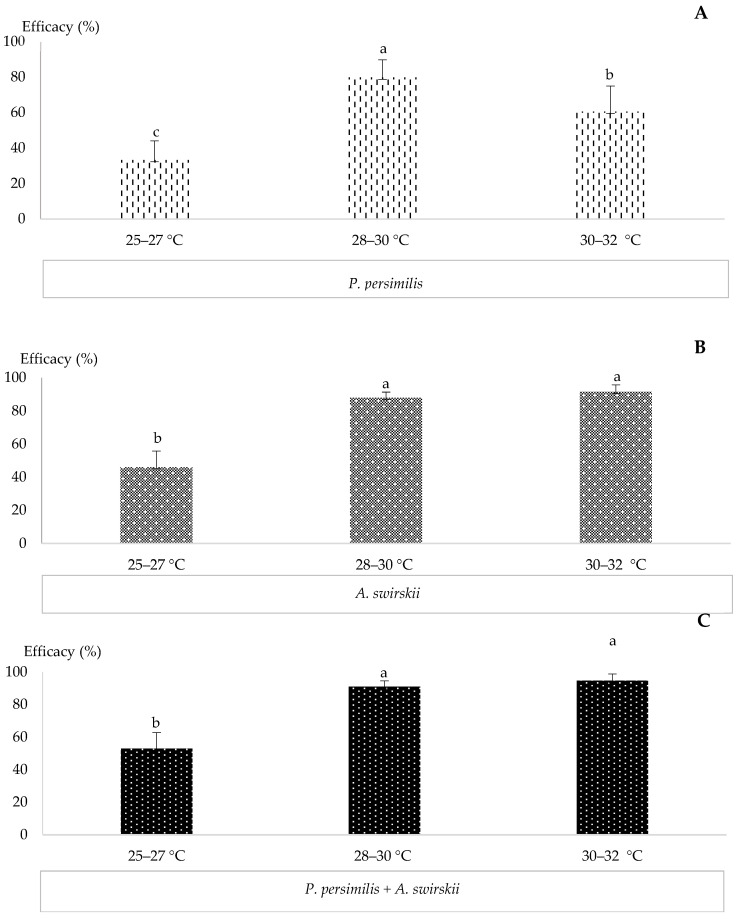
Efficacy of *P. persimilis*, *A. swirskii*, and their combined release in controlling *T. urticae* across different temperature ranges. ((**A**) Efficacy of *P. persimilis*, (**B**) Efficacy of *A. swirskii*, (**C**) Efficacy of the combined release of *P. persimilis* and *A. swirskii*; different lowercase letters indicate significant differences among the treatments and control according to Tukey’s test at *p* < 0.05).

The comparative efficacy of *P. persimilis*, *A. swirskii*, and their combined release in controlling *T. urticae* across three temperature ranges (25–27 °C, 28–30 °C, and 30–32 °C) is presented in Figure 6. For *P. persimilis*, the highest efficacy (79.77 ± 10.02%) was observed at 28–30 °C, showing a significant difference (*p* = 0.003) compared with its efficacy at 25–27 °C and 30–32 °C (Figure 6A). For *A. swirskii*, the highest efficacy (91.47 ± 4.2%) occurred at 30–32 °C, showing a significant difference (*p* = 0.0091) compared with its efficacy at 25–27 °C (Figure 6B).

The combined release of *P. persimilis* and *A. swirskii* was more effective at temperatures above 28 °C, with the highest efficacy (94.63 ± 3.12%) recorded at 30–32 °C. This result showed a significant difference (*p* = 0.0036) compared with its efficacy at 25–27 °C (Figure 6C).

The efficacy of *P. persimilis*, *A. swirskii*, and their combination in controlling *T. urticae* across temperature ranges of 25–27 °C, 28–30 °C, and 30–32 °C is presented in Table 1. At 25–27 °C, the combined treatment of *P. persimilis* and *A. swirskii* showed a numerically higher efficacy (52.98 ± 8.44%) compared to *P. persimilis* alone (33.28 ± 10.78%) or *A. swirskii* alone (45.98 ± 9.83%), although the difference was not statistically significant (*p* = 0.49). At 28–30 °C, the combined treatment again showed higher efficacy (91.00 ± 4.58%) compared to *P. persimilis* alone (79.77 ± 10.01%) or *A. swirskii* alone (87.84 ± 3.49%), yet differences remained non-significant (*p* = 0.160). At 30–32 °C, the combined treatment exhibited the highest efficacy (94.63 ± 3.10%), surpassing both *P. persimilis* (60.57 ± 14.39%) and *A. swirskii* (91.47 ± 4.26%), and was significantly more effective than *P. persimilis* alone (*p* = 0.001).

## 4. Discussion

The two-spotted spider mite, *T. urticae*, is one of the most significant pests affecting greenhouse-grown strawberries, negatively impacting both yield and fruit quality. Previous studies have shown that its development, survival, reproduction, and longevity are influenced by abiotic factors, with temperature being a key determinant of its reproductive and developmental rates [52,53,54,55,56].

In our experiment, the population of *T. urticae* per plant and per leaf increased with rising ambient temperatures, and a highly significant positive correlation was observed between temperature and mite population size on strawberry plants. These results are consistent with the findings of Praslicka and Huszar [52], who reported that temperature plays a crucial role in the development and multiplication rate of *T. urticae* populations. They observed that the mite develops fastest at 30 °C, completing its life cycle in 6.39 days, compared to 16.23 days at 15 °C. Similarly, Damos et al. [10] reported that higher temperatures and dry conditions shorten development time from egg to adult and increase female fecundity. Riahi et al. [55] indicated that *T. urticae* can develop and reproduce across a wide range of temperatures, with 27–30 °C being optimal for its development, survival, and reproduction. The positive correlation between temperature and mite population size is also supported by Meena et al. [56] and Adly [36].

In this study, following the establishment and population growth of *T. urticae* on strawberry plants, the predatory mites *P. persimilis* and *A. swirskii* were released either singly or in combination. The results demonstrated that both individual and combined applications of these predators successfully reduced *T. urticae* populations compared with the untreated control. However, the population dynamics differed between treatments. Unlike the single use of *A. swirskii* or its combination with *P. persimilis*, the *T. urticae* population curve showed a downward trend during the first six weeks after the release of *P. persimilis* alone, followed by an upward trend for the remainder of the experimental period. Overall, these results confirm that both predators are effective biocontrol agents, either individually or in combination, against the two-spotted spider mite. The effectiveness of *P. persimilis* against *T. urticae* is supported by studies conducted by Yanar et al. [24], Liao et al. [25], Bilbo and Walgenba [11], and Zhao et al. [2], which reported successful control of *T. urticae* on various greenhouse crops, including cucumbers, tomatoes, and strawberries, in several Mediterranean and Asian countries. The application of *A. swirskii* as a biocontrol agent against *T. urticae* is supported by Dalir et al. [29]. Furthermore, the effectiveness of combining *P. persimilis* and *A. swirskii* has been reported by Yasar et al. [28] and Abou-Haidar et al. [57]. The upward trend in the *T. urticae* population after six weeks in treatment with *P. persimilis* alone, compared with the single use of *A. swirskii* or their combined application, is likely due to the reduced efficiency of *P. persimilis* at higher temperatures.

Our results indicate that the efficiency of *P. persimilis* increases with temperature fluctuations between 25.33 and 28.2 °C, showing a highly significant positive correlation. When temperatures exceeded 28.2 °C, efficiency declined, and a significant negative correlation was observed, with peak efficiency recorded between 28 and 30 °C. These findings suggest that *P. persimilis* is most effective in controlling *T. urticae* within this temperature range, consistent with previous reports indicating optimal development at 27 °C and sensitivity to temperatures above 30 °C, ceasing feeding at approximately 35 °C [36,45].

Similarly, *A. swirskii* effectively controlled *T. urticae* under greenhouse conditions with temperatures ranging from 25.33 °C to 31.88 °C. Its efficacy increased with temperature, showing a highly significant positive correlation, with maximum effectiveness above 28 °C. These results are consistent with previous studies showing optimal survival and shortest development periods between 25 °C and 30 °C [32,57,58,59,60]. Developmental thresholds for *A. swirskii* were reported as 11.3 °C (lower), 37.4 °C (upper), and 31.5 °C (optimum) [57].

Overall, the combined release of *P. persimilis* and *A. swirskii* consistently provided the highest level of mite suppression across all temperature ranges (25–27 °C, 28–30 °C, and 30–32 °C), outperforming single-predator treatments. This approach effectively mitigates temperature-related variations in *T. urticae* populations. Previous studies corroborate the success of combined releases on sweet pepper, tomato, and rose plants [28,61,62]. However, simultaneous releases can lead to intraguild predation in the absence of sufficient prey, as *A. swirskii* may consume immature *P. persimilis* stages [63,64]. Therefore, the successful combined use of these predators requires adequate prey availability.

## 5. Conclusions

In conclusion, the present study demonstrates the effects of temperature on *T. urticae* populations in greenhouse-grown strawberry crop and on the efficacy of the predatory mites *P. persimilis* and *A. swirskii* when released using two methods: single-species and combined releases. The data indicate that higher temperatures accelerate the development of *T. urticae* population. Single-species releases of *P. persimilis* were effective at approximately 28 °C, whereas *A. swirskii* was effective at temperatures above 28 °C. The combined release of both predators was more effective throughout the experimental period, resulting in significantly greater population reduction at approximately 25–26 °C compared to single-species releases, with maximum efficacy observed at temperatures above 28 °C. These findings support the notion that combined releases of *P. persimilis* and *A. swirskii* provide more effective in controlling *T. urticae* and mitigate the effects of temperature fluctuations. The study was conducted under greenhouse conditions, where temperatures did not exceed 32 °C; however, some strawberry varieties continue fruiting until May or June, when temperatures may surpass 32 °C. Further research should investigate the effect of temperatures above 32 °C on the efficacy of combined releases of *P. persimilis* and *A. swirskii*. Additionally, the performance of predatory mites can be influenced by other abiotic factors, such as relative humidity. Therefore, future studies should also examine the impact of humidity on the efficacy of these predators when released alone or in combination.

## Figures and Tables

**Table 1:** Efficacy of *P. persimilis*, *A. swirskii*, and their combination for controlling *T. urticae* across three temperature ranges.

Efficacy (%)	Range of Temperatures		
	25–27 °C	28–30 °C	30–32 °C
*P. persimilis*	33.28 ± 10.78	79.77 ± 10.01	60.57 ± 14.39 ^b^
*A. swirskii*	45.98 ± 9.83	87.84 ± 3.49	91.47 ± 4.26 ^a^
*P. persimilis* + *A. swirskii*	52.98 ± 8.44	91.00 ± 4.58	94.63 ± 3.10 ^a^

Different lowercase letters within the column show significant differences between the treatments and control according to Tukey’s test at *p* < 0.05.

## Data Availability

The original contributions presented in this study are included in the article. Further inquiries can be directed to the corresponding author.
